# The Effect of Sleep Quality and Quantity on Athlete's Health and Perceived Training Quality

**DOI:** 10.3389/fspor.2021.705650

**Published:** 2021-09-10

**Authors:** Michael J. Hamlin, Richard W. Deuchrass, Peter D. Olsen, Maria A. Choukri, Helen C. Marshall, Catherine A. Lizamore, Claudia Leong, Catherine A. Elliot

**Affiliations:** ^1^Department of Tourism, Sport and Society, Lincoln University, Christchurch, New Zealand; ^2^Faculty of Health and Environmental Science, Sports Performance Research Institute New Zealand, Auckland University of Technology, Auckland, New Zealand; ^3^Recreation Centre, Lincoln University, Christchurch, New Zealand; ^4^Department of Applied Sciences and Social Practice, Ara Institute of Canterbury, Christchurch, New Zealand

**Keywords:** student-athlete, training stress, recovery, athlete monitoring, stress response, sport training

## Abstract

University athletes are unique because they not only have to cope with the normal psycho-physiological stress of training and playing sport, but they also need to accommodate the stress associated with their academic studies along with considerable stress from their social environment. The ability to manage and adapt to stress ultimately helps improve athletic performance, but when stress becomes too much for the athlete, it can result in maladaptation's including sleep disruption which is associated with performance loss, negative mood changes, and even injury or illness. This research aimed to determine if sleep quantity and quality were associated with maladaptation in university athletes. We examined subjective measures of sleep duration and sleep quality along with measures of mood state, energy levels, academic stress, training quality and quantity, and frequency of illness and injury in 82 young (18–23 years) elite athletes over a 1 year period in 2020. Results indicate sleep duration and quality decreased in the first few weeks of the academic year which coincided with increased training, academic and social stress. Regression analysis indicated increased levels of perceived mood (1.3, 1.1–1.5, Odds Ratio and 95% confidence limits), sleep quality (2.9, 2.5–3.3), energy levels (1.2, 1.0–1.4), training quality (1.3, 1.1–1.5), and improved academic stress (1.1, 1.0–1.3) were associated with ≥8 h sleep. Athletes that slept ≥8 h or had higher sleep quality levels were less likely to suffer injury/illness (0.8, 0.7–0.9, and 0.6, 0.5–0.7 for sleep duration and quality, respectively). In conclusion, university athletes who maintain good sleep habits (sleep duration ≥8 h/night and high sleep quality scores) are less likely to suffer problems associated with elevated stress levels. Educating athletes, coaches, and trainers of the signs and symptoms of excessive stress (including sleep deprivation) may help reduce maladaptation and improve athlete's outcomes.

## Introduction

Training is designed to produce stress beyond the body's ability to cope, which then sets in motion subsequent adaptation, resulting over time, in super-compensation, and a training effect if recovery is adequate. The key to efficient and effective exercise training is managing the training load with recovery. If training recovery is insufficient, stress can build up resulting in maladaptation and performance loss. On the other hand, too much recovery can result in insufficient stress, little adaptation, and little performance gain. The amount of recovery required to allow optimal restitution of bodily functions varies depending on the athletes physiological and psychological profiles (Bishop et al., 2008), the preceding training stimulus along with the athletes accumulated training stimuli. Moreover, recovery from athletic training can also be influenced by sleep (Bird, 2013).

There is a strong positive association between sleep and athletic performance including sports-specific skill execution, strength, and anaerobic power (Walsh et al., 2021). Sleep is a basic requirement for health and recovery that is believed to be related to homeostatic processes that rejuvenate and replenish major physiological and psychological functions of the human body (Lastella et al., 2015). There is ongoing controversy around how much sleep an athlete requires per night, with recent studies from the National Sleep Foundation suggesting that healthy adults should obtain anywhere between 7 and 9 h of sleep per night to carry out daytime functions (Sargent et al., 2014). Athletes are expected to have approximately 8 h of sleep per night to prevent the neurobehavioral deficits associated with sleep loss (Lastella et al., 2015).

Lack of sleep is shown to have detrimental effects on physiological and psychological performance (Leeder et al., 2012). The most prevalent effects of sleep loss are psychological, with the primary affect being associated with altered mood states, decision making skills, and cognitive impairment (Davenne, 2009). Decision-making skills are frequently incorporated into sport, and when sleep duration and sleep quality is not constantly prioritized, the cognitive processes involved in decision making during sport are impaired, thus decreasing performance outcomes (Reilly and Edwards, 2007). Physiological effects with sleep loss are not so prevalent but are linked to reduced immune function (via reductions in natural killer T cells) (Reilly and Edwards, 2007), decreased sub-maximal sustained performance (Leeder et al., 2012), and even reduced glucose metabolism (Spiegel et al., 1999) which may result in increased fatigue (Davenne, 2009).

Factors such as gender, and type of sport or exercise can all affect an athlete's sleep patterns, with some researchers suggesting that females have substantially better sleep quality than males of the same age range (Leeder et al., 2012). Contrary to this, others suggest that the effects of sleep deprivation are the same for females and males (Reilly and Edwards, 2007). Different sports also influence athletes sleep patterns with the combination of factors such as training volume and intensity, frequency of training, psychological stress of training (particularly with pre-competition training), and external factors such as work, family relationships, and academic commitments (Leeder et al., 2012). Differences in sport competitions and stages of training also accounts for variability in sleep patterns.

Therefore, the primary objective of this research was to examine the sleep patterns (sleep duration and quality) of young elite athletes in a university educational environment. We were particularly interested in how sleep patterns might change over the academic year. A secondary objective was to investigate the relationship between measures of perceived sleep duration (whether athletes attained at least 8 h sleep) as promoted by some researchers (Lastella et al., 2015; Roberts et al., 2019) and sleep quality with subjective measures of mood, energy levels, muscle soreness, academic stress, injury, and illness, and perceived training performance.

## Materials and Methods

### Subjects

Sleep duration and sleep quality, subjective measures of wellness along with training loads were retrospectively investigated in 82 young athletes during their academic year at university (typically February to October 2020). Students were involved in a university sport scholarship program where athletes received nutritional, psychological, and medical advice along with individualized training. All participants were young elite athletes (18–23 years old) selected for age-group regional or national representative honors. This study was carried out in accordance with the recommendations of the Lincoln University Human Ethics Committee. All subjects gave their written informed consent in accordance with the Declaration of Helsinki. The protocol was approved by the University's Human Ethics Committee (reference 2018-01). Participant characteristics are presented in Table 1. Body mass, reported in kg (to 1 decimal point) was measured on calibrated scales (Seca, 762, Hamburg, Germany) with the athlete's shoes and socks removed and in light training clothing. Height (to the nearest 0.1 cm) was measured using a portable stadiometer (Seca 213, Hamburg, Germany). The sum of eight skinfolds (bicep, triceps, subscapular, abdominal, supraspinale, iliac crest, front thigh, and medial calf) were taken using International Society for the Advancement of Kinanthropometry (ISAK) guidelines, by an ISAK-qualified level 3 practitioner (Norton et al., 1996). There was a wide cross-section of sports represented including rugby union (*n* = 28), netball (*n* = 11), basketball (*n* = 13), cricket (*n* = 8), field hockey (*n* = 13), athletics (*n* = 3), rowing (*n* = 2), and others (*n* = 4).

**Table 1:** Physical characteristics of athletes.

	**Male**	**Female**	**All**
	**(** ***n*** **= 55)**	**(** ***n*** **= 27)**	**(** ***n*** **= 82)**
Age (years)	19.8 ± 1.5	20.0 ± 1.4	19.9 ± 1.5
Height (cm)	185.3 ± 7.4	172.8 ± 6.6	181.5 ± 9.2
Body mass (kg)	88.6 ± 12.0	69.3 ± 7.2	82.2 ± 14.0
Skinfold thickness (mm)	84.0 ± 36.7	100.5 ± 27.9	89.6 ± 34.7

*Data are mean ± SD. Skinfold thickness; sum of eight sites (bicep, triceps, subscapular, abdominal, supraspinale, iliac crest, front thigh, and medial calf)*.

### Study Design

This longitudinal retrospective study used a commercially available software system (Health and Sport Technologies Ltd., trading as Metrifit, Millgrange, Greenore, Co. Louth, Ireland) to collect training data along with subjective feelings of mood, sleep quality/quantity, academic stress, and training performance in athletes during their time at university. The data was collected using the Metrifit phone application 2–3 weeks prior to the start of university during their orientation period, and then throughout the athlete's academic year. Each semester normally comprises of 12-weeks of teaching, a 1-week study break, followed by a 2-week final examination period to close the semester. Most students spend holidays (mid and end-of-semester) away from university, for example, returning home to spend time with their families or traveling. In 2020 however, because of the COVID-19 pandemic, the New Zealand government instituted a strict containment strategy from 25 March until 8 June where students were asked to leave the university and travel back to their homes and maintain studies via on-line learning. Therefore, the athletes in this study were at university for the first 6 weeks of semester 1 and returned home for 14 weeks (8 weeks semester break, holidays and exams, and 6 weeks of university work by on-line learning), before returning to on-campus university life for the whole of the second semester.

### Training

Individualized training programs were developed by the strength and conditioning staff at the university for each athlete, depending on the type of athlete, their competitive season, and injury status. In most weeks, athletes would have at least three training sessions, one sport-specific skills session and one practice game or competition (except in the COVID-19 lockdown when games and competitions were substituted with training sessions). Athletes recorded their daily training information including type, duration, and intensity of training. The intensity of training was estimated using a modified 10-point scale (Foster et al., 2001). Previous research by our group (Hamlin and Hellemans, 2007) and others (Eston and Williams, 1988; Impellizzeri et al., 2004; Gabbett and Domrow, 2007), support these effort ratings as reliable indicators of exercise intensity.

The training load (internal training load) was calculated as the product of volume (duration of training) and intensity (subjective rating of training intensity) as proposed by Foster et al. (2001). It is well-documented that subjective measures (mood disturbance, perceived stress, sleep disruption, etc.) consistently show superior responsiveness to training compared to objective measures (Verde et al., 1992; Coutts et al., 2007; Saw et al., 2016). Unfortunately many existing subjective questionnaires [e.g., Recovery Stress Questionnaire for Athletes (Kellmann and Kallus, 2001), Daily Analysis of Life Demands of Athletes (Rushall, 1990), and Multi-Component Training Distress Scale (Main and Grove, 2009)] are long with numerous questions making them time-consuming and complicated and not fit for purpose in a practical setting. Because of this, the Lincoln University Sport Scholarship program decided to incorporate elements of established measures into our own customized, brief, easy-to-use, self-reported measures. For this study we asked a series of questions used successfully in a number of other studies (Hamlin and Hellemans, 2007; Hamlin et al., 2017) which were modeled on previous research (Mackinnon and Hooper, 1996; Killen et al., 2010). The questions used in the phone App were based on a five-point Likert scale to record athlete's subjective ratings of mood, sleep quality, energy levels, muscle soreness, academic pressure, and perceived training quality (Table 2). Athletes were asked to complete the subjective data entry in the App a minimum of three times per week, where they had to move an electronic slider (which was initially situated on the far bottom of the screen, or at number “1” for each question) to the appropriate perceived subjective rating for the day for that question. Athletes also recorded their perceived sleep duration in hours and minutes from the previous night at the same time. The phone App also allowed the athletes to input descriptors of any illness or injury they may have, so these details were also collected over the period of the study. All athletes were given clear instructions on how to use the Metrifit system which included a 2-h training session around understanding the data required by the system and how to enter the data using the Metrifit App Interface on each student's phone. Athletes were encouraged to use the software to input data and they received email reminders on their mobile phones if data entry was missed. From the 82 athletes, data was gathered on 10,452 combined days, with each athlete completing data entry on an average of 127 out of ~270 days, with females completing slightly more (139 days) compared to males (121 days).

**Table 2:** The subjective measures used in the study.

	**Likert scale**				
**Subjective measures**	**1**	**2**	**3**	**4**	**5**
Mood state	Very stressed	Quite stressed	Slightly stressed	Little stress	No stress
Sleep quality	Poor	Below average	Normal	Good	Very good
Energy levels	Extremely low	Very low	Low	Normal	High/excellent
Muscle soreness	Extremely sore	Very sore	Quite sore	Mild soreness	No soreness
Academic pressure	Academic pressure high	Academic pressure building	Heavy academic day	Normal academic pressure	No academic pressure
Perceived training quality	Poor	Fair	Good	Very good	Excellent

While self-reported subjective questionnaires have been shown to overestimate athletes sleep compared to more objective measures (Carter et al., 2020), the use of objective measures such as polysomnography or actigraphy can be intrusive and require specialist staff, which can be inconvenient, expensive, and impractical when large numbers of athletes are involved in longitudinal studies. Therefore, in this study we have chosen to use simple subjective measures of sleep.

It is important to not only focus on current training regimes, but also what athletes have previously completed in terms of preparation for training. Previous work suggests a sharp increase in current training (acute training load), without the appropriate preparation (chronic training load), can result in injury (Gabbett, 2016). We therefore calculated the acute:chronic workload which gives an estimate of the preparedness of athletes to handle increases in workload stress using an exponentially weighted moving average (EWMA) as proposed by Williams et al. (2017). The calculation is as follows:


$$\begin{array}{l} {\text{EWMA}}_{\text{today}}=Load_{\text{today}}\times \lambda_{\text{a}}+\left(\left(1-\lambda_{\text{a}}\right)\times \;{\text{EWMA}}_{\text{yesterday}}\right) \end{array}$$

Where λ_a_ is a value between 0 and 1 representing the degree of decay, which assigns a lower weighting for older observations. The λ_a_ was calculated as:


$$\begin{array}{l} \lambda_{\text{a}}=2/\left(N+1\right) \end{array}$$

Where *N* is the chosen time decay constant in days, which was selected as 1-week (to represent acute workload over the last 7 days) and 4-weeks (representing chronic workload over the last 28 days). After arbitrarily recording the first observation in the dataset as the first observation, the above formula was used to calculate the average acute and chronic workloads for each week for all subjects combined. The acute:chronic ratio was then calculated by dividing the acute workload by the chronic workload (Williams et al., 2017).

### Statistical Analysis

Changes in the mean of the variables and standard deviations representing the between-and within-subject variability were estimated using a mixed modeling procedure (PROC MIXED) in the Statistical Analysis System (Version 9.4, SAS Institute, Cary North Carolina, USA). To investigate the effect of sleep duration, the daily perceived sleep duration data was separated into two groups (<8 h and ≥8 h) for further analysis. We also divided the sleep quality daily Likert score into two separate groups for further analysis (poor quality sleep Likert scores <3, good quality sleep Likert scores ≥3). Chances that the true effects were substantial were estimated when a value for the smallest worthwhile effect was entered into the calculation. We chose 0.20 standardized units (representing change in mean divided by the between-subject SD at baseline) as the smallest worthwhile change (Cohen, 1988). To make inferences about the true (population) uncertainties in the estimate of change were presented as 95% confidence intervals and as likelihoods that the true value of the effect was increased, decreased or trivial. The descriptors: increased, trivial, or decreased were used to describe the direction of the change. Where the confidence interval spanned all three possibilities (increased, trivial, and decreased), the result was deemed unclear. In all other cases, such as no overlap, or an overlap between two possibilities (trivial and increased, or trivial and decreased) a clear result was achieved. The magnitude or probability of the change were assigned qualitative descriptors defined as: <0.5%, almost certainly not; <5%, very unlikely; <25%, unlikely/probably not; 25–75%, possibly, possibly not; >75%, likely, probably; >95%, very likely; and >99.5%, almost certainly (Batterham and Hopkins, 2006). Individual subjective measures (mood state, sleep quality, energy levels, muscle soreness, academic pressure, perceived training quality), along with sleep duration (<8 or ≥8 h) and sleep quality (<3 or ≥3 on the Likert scale) were modeled together using an ordinal logistical regression (PROC LOGISTIC). We also performed logistical regression on sleep duration and sleep quality with incidence of illness and injury. The summary statistic used for assessing the adequacy of the fitted model (goodness of fit) was the likelihood ratio chi-square. Odds ratios (and 95% confidence limits) were calculated to determine whether changes in subjective measures as well as illness/injury were associated with sleep duration and quality. We also calculated the effect size statistics (ES, Cohen's *d*) from the change in the mean between groups divided by the between-participant SD. The magnitude of the effect size was interpreted using Hopkins et al. (2009) descriptors (i.e., 0.2 small, 0.6 moderate, 1.2 large, 2.0 very large) (Hopkins et al., 2009). Finally, we have also given the *p*-values for all analyses.

## Results

### Sleep Quantity and Quality Over a Week

**Figure 1:**
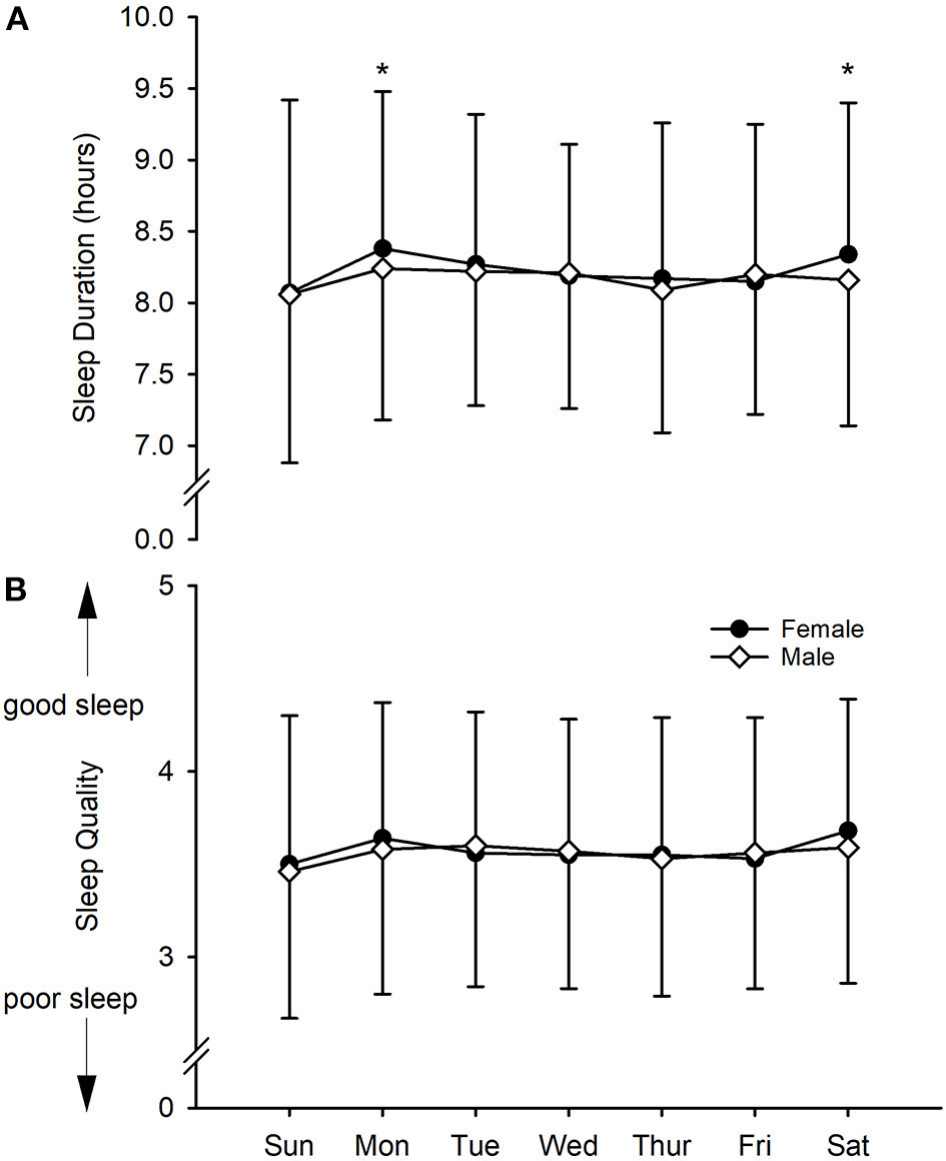
Subjective measures of young elite university athletes. **(A)** Sleep duration, **(B)** sleep quality. Values are daily means with SD as error bars. *Substantially different between male and female.

On average, male athletes slept for 8.2 ± 1.0 h/night (mean ± SD) while female athletes slept on average 8.2 ± 1.1 h/night. Similarly, average perceived sleep quality was comparable between female and male athletes (3.6 ± 0.8 and 3.6 ± 0.8, respectively). When separated into days of the week (Figure 1), we found that females and males tended to show similar sleep duration and quality patterns throughout the week except Monday and Saturday when females showed small increases in sleep duration compared to males (*ES* = 0.14 and 0.17, *p* = 0.01 and 0.01, respectively).

### Sleep Quantity and Quality Over a Year

**Figure 2:**
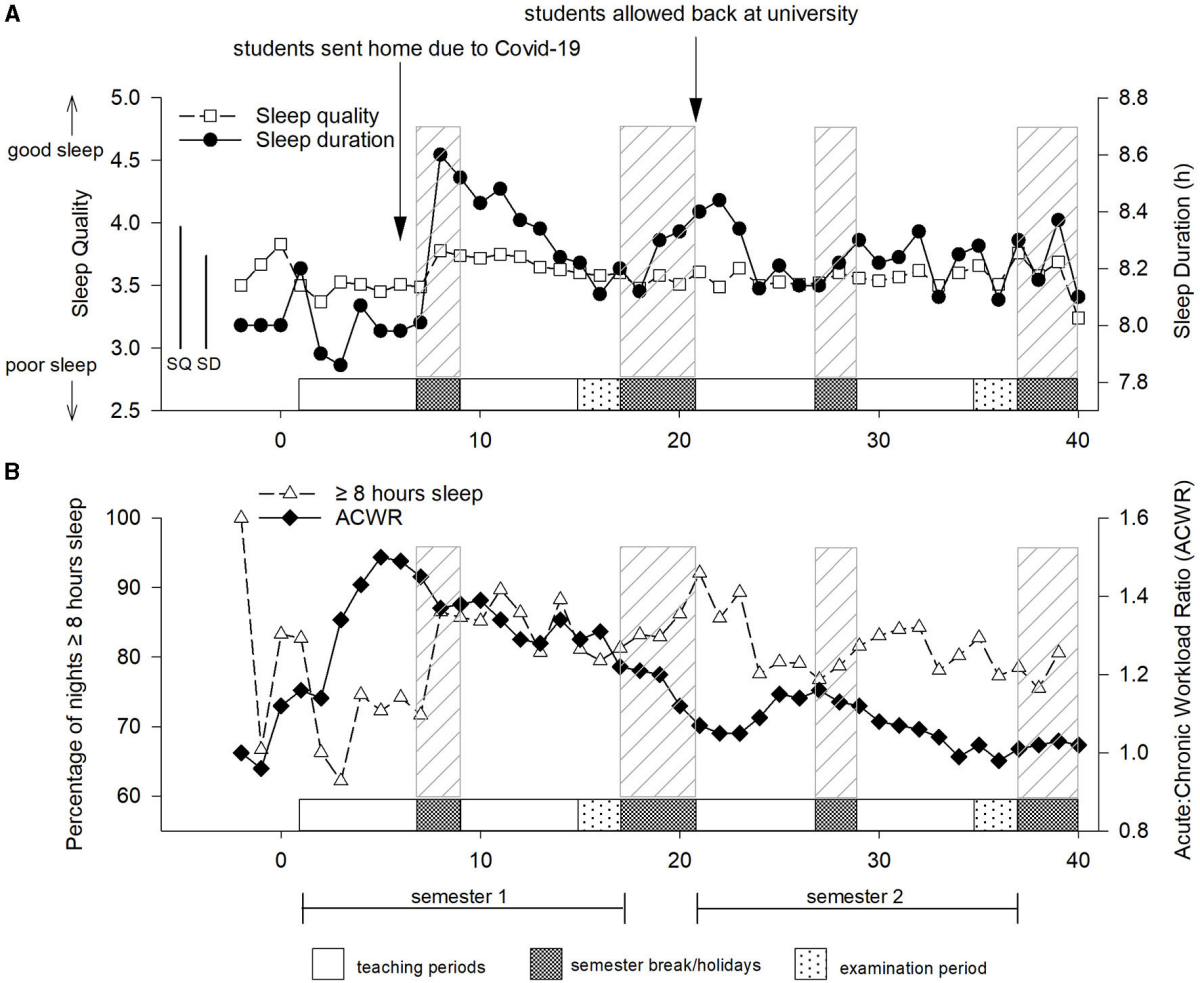
**(A)** Perceived sleep quality and duration, **(B)** percentage of nights slept ≥ 8 h and Acute:Chronic workload ratio (ACWR) in young elite university athletes during the university year. Values are weekly means. SQ, Sleep quality overall between-subject standard deviation; SD, Sleep duration overall between-subject standard deviation.

Sleep duration during the year fluctuated around 8 h per night until the athletes were sent home due to the COVID-19 pandemic (Figure 2A). Once athletes returned to university in the second semester, sleep duration was maintained between 8.1 and 8.4 h/night. Perceived sleep quality was at its highest (3.8 ± 0.4) the week prior to coming to university, and while at university, perceived sleep quality was maintained at approximately 3.5 throughout the year. Prior to the start of university, athletes reported sleeping for at least 8 h/night on all recorded nights (100%, see Figure 2B), which reduced to 62% on recorded nights in week 3. Overall, athletes reported sleeping ≥8 h/night on 81% of the recorded nights (males 79%, females 82%).

### Effect of Sleep Duration on Subjective and Training Measures

Table 3 indicates the differences in the subjective and training measures on days when athletes reported <8 h sleep compared to days when athletes reported eight or more hours sleep. On days when male athletes slept ≥8 h, subjective markers of mood state (*ES* = 0.34, *p* < 0.0001), sleep quality (*ES* = 0.85, *p* < 0.0001), and energy levels (*ES* = 0.46, *p* < 0.0001), showed small to moderate improvements, while all other measures showed trivial changes. Similarly in females, compared to days with <8 h sleep, on days when females had ≥8 h sleep, subjective markers of mood state (*ES* = 0.30. *p* < 0.0001), sleep quality (*ES* = 0.93, *p* < 0.0001), and energy levels (*ES* = 0.34, *p* < 0.0001) all showed small to moderate improvements. In addition, in females training quality showed a small improvement with longer sleep (*ES* = 0.27, *p* < 0.0001). All other changes were trivial.

**Table 3:** Subjective and training measures in young elite university athletes on days when they slept for <8 h compared to days when slept for 8 or more h.

	**Males (** ***n*** **= 55)**				**Females (** ***n*** **= 27)**			
	**<8 h**	**≥8 h**	**ES**	***P*-value**	**<8 h**	**≥8 h**	**ES**	***P*-value**
	**(** ***n*** **= 1,137)**	**(** ***n*** **= 4,624)**			**(** ***n*** **= 746)**	**(** ***n*** **= 2,578)**		
Mood state	3.7 ± 0.6	3.9 ± 0.5	0.34	<0.0001	3.7 ± 0.6	3.9 ± 0.5	0.30	<0.0001
Sleep quality	3.1 ± 0.8	3.7 ± 0.7	0.85	<0.0001	3.0 ± 0.8	3.7 ± 0.7	0.93	<0.0001
Energy levels	3.5 ± 0.7	3.8 ± 0.6	0.46	<0.0001	3.6 ± 0.6	3.8 ± 0.5	0.34	<0.0001
Muscle soreness	3.7 ± 0.7	3.8 ± 0.7	0.10	0.03	4.0 ± 0.7	3.9 ± 0.6	0.10	0.013
Academic pressure	3.7 ± 0.9	3.8 ± 0.8	0.10	0.0007	3.8 ± 0.9	3.9 ± 0.8	0.10	0.01
Training quality	3.2 ± 0.7	3.3 ± 0.6	0.10	0.05	3.2 ± 0.7	3.4 ± 0.6	0.27	<0.0001
Training duration (min)	79.3 ± 40.8	82.0 ± 39.2	0.06	0.22	71.3 ± 35.6	73.4 ± 39.1	0.05	0.40
Training RPE	5.9 ± 1.7	5.8 ± 1.6	0.06	0.36	5.5 ± 1.8	5.7 ± 1.6	0.13	0.04
Training load (au)	480.1 ± 315.4	486.0 ± 281.1	0.02	0.71	391.1 ± 238.8	426.9 ± 279.0	0.12	0.04

*Data are mean ± SD. RPE, rate of perceived exertion; au, arbitrary units, ES, effect size. Sleep duration ranged from 1.0 to 7.8 h for the <8 h data and 8.0 to 14.0 h for the ≥8 h data*.

### Logistical Regression Outcomes

Outcomes from the ordinal logistical regression are shown in Table 4 and indicate improved mood (lower stress), improved sleep quality, energy levels, and increased perceived training quality were associated with ≥8 h sleep. The model was successful at fitting the data as evidenced by the likelihood ratio χ^2^ = 393.5 with nine degrees of freedom, *p* < 0.001. The final model was −4.4076 + 0.2451 ^*^ mood + 1.0640 ^*^ sleep quality + 0.1402 ^*^ energy – 0.1882 ^*^ muscle soreness + 0.1211 ^*^ academic + 0.00405 ^*^ training duration – 0.0220 ^*^ training RPE – 0.00030 ^*^ total training load + 0.2457 ^*^ perceived training quality.

**Table 4:** Association between sleep characteristics and athlete's subjective and training parameters.

**Parameter**	**Odds ratio (95% CL)**
**Sleep Duration (<8 h vs**. **≥8 h)**	
Mood	1.3 (1.1–1.5)
Sleep quality	2.9 (2.5–3.3)
Energy	1.2 (1.0–1.4)
Muscle soreness	0.8 (0.7–1.0)
Academic	1.1 (1.0–1.3)
Training duration	1.0 (0.9–1.0)
Training RPE	0.9 (0.9–1.1)
Total training load	1.0 (0.9–1.0)
Perceived training quality	1.3 (1.1–1.5)
**Sleep Quality (<3 vs**. **≥3 on Likert Scale)**	
Mood	1.2 (0.9–1.5)
Sleep duration	3.3 (2.8–3.9)
Energy	3.0 (2.3–3.9)
Muscle soreness	0.9 (0.7–1.1)
Academic	0.8 (0.7–1.0)
Training duration	1.0 (0.9–1.0)
Training RPE	1.0 (0.9–1.1)
Total training load	1.0 (1.0–1.0)
Perceived training quality	0.9 (0.7–1.1)

*Data is odds ratio (95% confidence limits). RPE, rate of perceived exertion*.

Improved mood, higher sleep duration, and higher energy levels were also associated with better sleep quality (Table 4). The model was successful at fitting the data as evidenced by the likelihood ratio χ^2^ = 434.4 with nine degrees of freedom, *p* < 0.001. The final model was −9.857 + 0.1456 ^*^ mood + 1.2067 ^*^ sleep duration + 1.0971 ^*^ energy – 0.0883 ^*^ muscle soreness – 0.1927 ^*^ academic – 0.00098 ^*^ training duration – 0.0217 ^*^ training RPE + 0.00038 ^*^ total training load – 0.1335 ^*^ perceived training quality.

### Injury and Illness

Leg injuries were the most commonly reported injury, whereas colds and influenza made up the majority of the illnesses reported (Table 5). Completing an ordinal logistical regression between sleep duration and sleep quality with injury/illness reporting, we found that when athletes slept ≥8 h per night they were less likely to suffer injury/illness (OR = 0.8, 95% CI = 0.7–0.9, model = −2.6282 – 0.2154 ^*^ sleep duration, likelihood ratio χ^2^ = 4.0096 with one degree of freedom, *p* = 0.04). Similarly, higher the sleep quality reduced the odds of injury/illness (OR = 0.6, 0.5–0.7, model = −1.0599 – 0.5053 ^*^ sleep quality, likelihood ratio χ^2^ = 76.24 with one degree of freedom, *p* < 0.0001). All other associations were trivial.

**Table 5:** Injury and illness reported by the athletes during the study.

**Injury area**	**Illness type**
Arm (7)	Chest infection (2)
Back (28)	Cold/Influenza (192)
Chest (3)	Diarrhea (5)
Foot/Ankle (32)	Migraine/Headaches (37)
Groin (6)	Nausea/Vomiting (7)
Leg (110)	Other (37)
Neck (8)	
Shoulder (37)	
Other (22)	

*Data is injury area and illness type along with frequencies of these injuries/illnesses in brackets*.

## Discussion

The aim of the present study was to investigate how perceived sleep patterns (duration and quality) change during the year in a cohort of young elite university athletes and to what extent perceived sleep duration and quality affects subjective markers of training performance and wellness. Sleep has been found to facilitate important recovery-enabling functions including recovery of the physiological, psychological, musculoskeletal, immune, metabolic, and endocrine systems, all of which are required for athlete's successful adaptation to training stress. Some authors propose that sleep is associated with recovery and performance in athletes (Walsh et al., 2021) and is closely associated with the stress response. The main results of this study showed the following (i) perceived sleep duration and quality were similar in male and female athletes except females slept longer on Monday's and Saturday's, (ii) perceived sleep duration and quality were negatively affected to a greater extent at the start of the academic year, (iii) longer sleep duration (≥8 h/night) and better quality sleep (Likert ≥3) was positively associated with subjective markers of mood and energy levels, (iv) sleeping ≥8 h/night or improved sleep quality reduced the athletes risk of injury/illness.

Previous work has indicated that the start of the academic year is generally a period of increased stress for athletes and students (Galambos et al., 2009; Hamlin et al., 2019). According to Nixdorf et al. (2015), stress for student-athletes comes from three areas; (i) double-burden stress; from combining sport and other duties, in this case education, (ii) sport-specific stress; the psycho-physiological stress associated with sport participation, and (iii) conditional stress; which is stress from unfavorable structures within the team/environment (Nixdorf et al., 2015). Sleep disruption is a commonly reported symptom of increased stress in athletes particularly through increased training workload (Kellmann, 2010) or intensified training blocks (Killer et al., 2017; Hoshikawa et al., 2018; Hrozanova et al., 2019). Indeed decreased sleep quantity and quality at the beginning of the university year, in the athletes reported in this study, coincided with the highest acute:chronic workloads suggesting increased training stress may be associated with the disturbed sleep. Some researchers suggest poor sleep is associated with two sleep-disturbing phenomena; (i) preservative cognition (e.g., worry or anxiety about performance or success) and (ii) hyper-arousal (increased neurobiological and psychological stresses during sleep) (Hrozanova et al., 2021). Therefore, the increased arousal that occurs with intensified training, or the anxiety associated with training and performance may result in disturbed sleep in athletes (Carney and Waters, 2006). Interestingly, daily use of cryotherapy, which is known to induce parasympathetic reactivation (Al Haddad et al., 2010) increased sleep quantity in elite swimmers undergoing intense training (Schaal et al., 2015) which suggests promoting relaxation may be a useful strategy for reducing sleep disturbance in athletes. Figure 2B illustrates that student athletes in this study are particularly susceptible to sleep disruption at the beginning of the year which coincides with a number of potentially stressful events at this time (i.e., meeting new people, new academic workload, and increased training load), which together with anxiety about performance may increase arousal and thereby reduce sleep. Once the academic year was underway (after about week 6–7), when presumably athletes were accustomed to training and were more relaxed about academic and social pressures, the athletes reported sleeping for ≥8 h per night about 80–90% of the time. Therefore, it would seem that interventions to mitigate disturbed sleep in these athletes should be focused on the start of the academic year when athletes have a number of stresses to deal with.

Higher mental stress and anxiety has also been associated with sleep disturbance in athletes (Hrozanova et al., 2020), whereas athletes with higher mental resilience tend to have longer sleep duration (Hrozanova et al., 2019). The results of the current study indicate a small positive relationship between athletes that reported sleeping longer (≥ 8 h/night), or having a better quality sleep and lower levels of stress (mood state) than those that slept <8 h/night or had a poorer sleep (Likert level <3). Kalmbach et al. (2018) suggests a cyclic effect whereby increased worry/anxiety (preservative cognition) in the athlete activates heightened arousal (hyper-arousal) that is incompatible with sleep, and that the inability to sleep when stressed results in more time worrying and therefore more sleep disturbance (Kalmbach et al., 2018). Reducing worry and anxiety in athletes with the help of psychological interventions may help to reduce overall stress and improve sleep.

Increased sleep duration (≥8 h/night) was associated with improved sleep quality and a trivial improvement in academic pressure in the athletes of this study. A number of previous reports have shown that insufficient or poor-quality sleep is associated with poor academic performance (Singleton and Wolfson, 2009; Taylor et al., 2013) and students with sleep disorders had increased risk of academic failure (Gaultney, 2016). It is thought that athletes that have too much stress or cannot respond to stress in an appropriate way have a maladaptive response to stress and become poor sleepers (Hrozanova et al., 2021), while those that can adapt appropriately, respond better, and sleep well. While the overall yearly data showed only trivial relationships between sleep duration and quality with academic pressure (Table 4), when we analyzed the relationship by each semester a much stronger association was evident between sleep quality and academic pressure in semester 1 (OR = 0.7, 0.6–0.9) when the athletes were under more stress particularly at the beginning of the semester. The double-burden of academic and athletic stresses at some times of the academic year may be too much for the athlete (particularly at the start of the year), which results in maladaptation and poor sleep quality and quantity.

Regression analysis indicated that on days when athletes reported ≥8 h sleep, or had a higher quality sleep, energy levels were more likely higher. Sleep is important for physiological recovery, and poor sleep is associated with increased perceived effort, delayed reaction time, mood disturbance, and reduced time to failure (Taheri and Arabameri, 2012; Fullagar et al., 2015; Watson, 2017). Better sleep may allow more recovery which may improve training quality and energy levels.

Days when athletes slept longer (≥8 h), or reported higher quality sleep were associated with a significantly reduced chance of injury and illness. A lack of adequate sleep is associated with reduced reaction times, altered cognitive functions, and mood state which may increase the risk of injury (Philip et al., 2004; Durmer and Dinges, 2005). Disturbed sleep is also associated with increased physical stress which can increase muscle tension resulting in altered motor coordination and flexibility thereby influencing fatigue and possibly injury (Williams and Andersen, 1998). Too much stress in athletes can also decrease immunity; for example heavily trained athletes with signs of sleep disturbance reported more upper respiratory tract infections (Hausswirth et al., 2014). However, despite these findings, the relationship between training loads, recovery, sleep, injury, and performance are complex, poorly understood, and are probably sport-specific and individual, thereby necessitating individualized approaches for athletes.

Interestingly, in females on days where they reported increased sleep (≥8 h), they also reported a trivial to small increase in perceived exertion during training (*p* = 0.04) and a small increase in overall training workload (*p* = 0.04). We also found a non-significant increase in total training workload in males on days when they slept ≥8 h and we suspect that on such days, these athletes have facilitative responses to the overall stress, allowing adaptation and a good sleep (Hrozanova et al., 2021). Or conversely, on days when athletes reported less sleep (<8 h) the overall stress was potentially too much for the athletes to cope with, resulting in maladaptation resulting in poor sleep.

We acknowledge the following limitations of the present study. The current study used subjective measures of health and wellness as well as perceived measures of sleep quantity and quality. While these measures are quick and easy to gather, they have not been validated by more robust measures (for example actigraphy). However, while the absolute numbers may not correspond exactly to objectively derived figures, we are confident the relative trends shown in this study are valid and show useful information for practical application in the field. Secondly, our study was completed on athletes in a rage of sports that all have different seasonal requirements and training schedules. While the bulk of the athletes were in winter sports (i.e., rugby, hockey, netball, basketball), we also had some athletes that were in summer sports (i.e., cricket), therefore pre-season, in-season and off-season would have been different between sports. However, because we have investigated the data on a day-by-day basis (i.e., not split into pre-season etc.) the problem of different training seasons is reduced. Thirdly, the present study included athletes from a relatively young population from a single university, which decreases the generalizability to the broader athlete populations. Finally, we must mention the fact that COVID-19 occurred during this study, which resulted in the lockdown of the country for a number of weeks, along with the athletes returning back to their homes. While the athletes maintained their training schedules, this unusual event may have affected mental and psychological stress within the athletes. It would be interesting to complete the study again in a year when we did not have a pandemic to see if results are similar to this study.

## Conclusion

Elite young athletes undertaking university studies have unique stressors including the double-burden of performing on the sports field and in the classroom, sport-specific stressors such as the psycho-physiological stress associated with their sport participation, and conditional stressors which might involve stress from within the team or external environment. If resilient enough, athletes will demonstrate facilitative reactions to such stress thereby positively adapting resulting in longer and higher quality sleep, improved mood state, higher energy levels, lower academic pressure, and a higher perceived training quality. On the other hand, when such stressors overwhelm the athletes coping ability (for example at the start of the academic year with stress from sport, academia and the athlete's social life), maladaptation ensues, resulting in less sleeping duration, poorer quality of sleep, reduced subjective feeling of wellness, and increased risk of illness and injury.

## Data Availability Statement

The dataset for this article is not publicly available because of commercial sensitivity in regards to the software system used in collecting the data (Health and Sport Technologies Ltd, trading as Metrifit, Millgrange, Greenore, Co. Louth, Ireland). Requests to access the datasets should be directed to Michael J. Hamlin (mike.hamlin@lincoln.ac.nz).

## Ethics Statement

The studies involving human participants were reviewed and approved by Lincoln University Human Ethics Committee, Lincoln University, Christchurch, New Zealand. The patients/participants provided their written informed consent to participate in this study.

## Author Contributions

MH conceptualized and designed the study. MH and RD, assisted in the planning and acquisition of data. MH, RD, PO, MC, HM, CAL, CL, and CE helped with the analysis and interpretation of the data, critically revising the manuscript, and added important intellectual content. All authors gave approval for the final version of this manuscript to be published and agree to be accountable for all aspects of the work.

## Funding

No financial or material support of any kind was received to produce the work described in this article, however the publication has been partially financed by the Lincoln University Open Access Fund.

## Conflict of Interest

The authors declare that the research was conducted in the absence of any commercial or financial relationships that could be construed as a potential conflict of interest.

## Publisher's Note

All claims expressed in this article are solely those of the authors and do not necessarily represent those of their affiliated organizations, or those of the publisher, the editors and the reviewers. Any product that may be evaluated in this article, or claim that may be made by its manufacturer, is not guaranteed or endorsed by the publisher.
